# Postpartum Amenorrhoea among Manipuri Women: A Survival Analysis

**DOI:** 10.3329/jhpn.v30i1.11288

**Published:** 2012-03

**Authors:** N. Sanajaoba Singh, N. Sharat Singh, R.K. Narendra

**Affiliations:** ^1^Directorate of Census Operations, Porompat, Manipur, India; ^2^Thoubal College, Thoubal, Manipur, India; ^3^Unit of Biostatistics, Regional Institute of Medical Sciences, Imphal, India

**Keywords:** Breastfeeding, Community-based studies, Cross-sectional studies, Infant mortality, Parity, Postpartum amenorrhoea, India

## Abstract

Among the three major components of a closed birth interval, waiting time to conception can somehow be managed with effective contraceptives while gestation is universally constant in its duration; the duration of postpartum amenorrhoea (PPA) varies in complex nature. The present study aimed to investigate the proximate factors influencing the duration of PPA. A community-based, cross-sectional study was conducted in four valley districts of Manipur, India, during 1 August−31 December 2009, to analyze the differentials and determinants of duration of PPA, applying the survival analysis technique. In total, 1,225 ever-married women were selected through two-stage cluster sampling. The median duration of PPA was 5.7 months. Among the 11 explanatory variables of interest, only three variables—place of residence (p<0.05), infant mortality from preceding pregnancy (p<0.01), and duration of breastfeeding (p<0.01)—had a significant effect on the duration of PPA. The findings may be used as baseline information for future researchers and maternal health policy-makers.

## INTRODUCTION

The duration of postpartum amenorrhoea (PPA) is the length of time between the termination of pregnancy and the first successive ovulation following the pregnancy during the reproductive span of a woman. In other words, it is the temporary sterility immediately following the termination of pregnancy during which conception usually does not occur. The attainment of first menstruation after delivery is treated as the termination of PPA. Being a physiological process associated with each conception, the importance of PPA in regulating human fertili-ty has been a matter of general interest for many years because it tends to increase the average birth interval, thereby reducing women's fertility over her life span, especially in societies where the use of contraceptive methods is not widespread. There is substantial evidence that long-term breastfeeding is associated with a longer anovulatory period leading to prolonged PPA. It also reduces fertility even after menses and ovulation have returned. Prolonged lactation suppresses the production of certain types of hormones, thereby extending the postpartum anovulatory period. Moreover, there are different social norms, taboos, and other socioeconomic factors, especially in traditional societies, that reinforce the effect of breastfeeding. However, many mechanisms by which breastfeeding behaviour and other factors produce variability in the length of the anovulatory period and thus, in the resumption of menses across populations and within a population in different social and cultural groups remain either unspecified or unknown. They are probably associated with the biological characteristics and specific social structures and implicit or explicit social norms.

The anovulatory period depends on a number of other factors, which vary from woman to woman in a population (1,2). PPA is associated with age of mother, marital duration, nutritional status, and parity (3-7). In these studies, breastfeeding is also found to be associated with delayed return of menstrual cycles and ovulation, reduced fecundability, and long birth intervals. The most important conclusion is that long-term breastfeeding was statistically associated with a long period of PPA (6,8-11). In the mid-1970s, some pioneering studies also stressed that, at least at the aggregate level, when regressing the average durations of PPA on the average durations of breastfeeding, the two are significantly and positively related (12,13). However, in the absence of breastfeeding, the average amenorrhoeic period may last 1-3 months (11,12). Education and family income have been inversely related with the duration of PPA (7,10,13).

In the policy point of view, the present empirical study was conducted in Manipur, one of the northeastern states of India. The study was conducted to investigate the nature and pattern of duration of PPA and to check the relative influences of various sociodemographic factors on it. The findings may be of immense values, particularly in couple's reproductive management, population planning, and health policy through which an attempt may also be made to improve the sociodemographic status of women in Manipur. Besides, the work is of considerable importance from the academic and application points of view, and as such, it assumes great significance as baseline information of reproductive health.

## MATERIALS AND METHODS

### Data

This community-based, cross-sectional study was conducted in four valley districts of Manipur, namely Bishnupur, Thoubal, Imphal West, and Imphal East, during 1 August—31 December 2009 using the two-stage cluster-sampling technique, with 11 July as the reference date of survey. The study subject was defined to be an eligible woman having at least one livebirth. The pretested and semi-structural type of interview schedule was used as the tool for collecting the required information. The sample consisted of 1,225 eligible women. The sample-size was determined based on the findings of Sharat (14), i.e. the mean±standard deviation (SD) of duration of closed birth interval is 21.05±18.79, under 95% degree of precision, with 5% margin of error to the mean (14). Altogether, clusters of 45 rural villages and 35 clusters of urban wards were randomly selected, of which 5 villages and 7 wards, 9 villages and 10 wards, 11 villages and 12 wards, and 20 villages and 6 wards were, res-pectively, selected proportionally from Bishnupur, Thoubal, Imphal West, and Imphal East districts. Of the 1,225 eligible women, 180, 316, 387, and 342 were randomly picked up from the selected clusters of Bishnupur, Thoubal, Imphal West, and Imphal East districts respectively.

### Response variable

The duration of PPA was taken as the response variable. Since ovulation itself is difficult to identify, the reliable estimate of the end of amenorrhoea is the return of menstruation itself. In this study, the duration of PPA was estimated as an interval between the termination of conception and the return of first menses. Women reporting the termination of amenorrhea before the survey date were considered uncensored cases, and their duration of PPA was the interval between the termination of conception and the return of the first mense. On the other hand, women reporting continuance of amenorrhoea even after the survey date were considered censored cases, and their event history of interest was the interval between the termination of conception and the survey date. In the case of women having more than one livebirth, the duration variable, i.e. the duration of PPA for the last birth, was taken into consideration to control the data recall error.

### Explanatory variables

The explanatory variables or so termed as covariates were demographic and socioeconomic factors. The socioeconomic variables included the place of residence, educational level, and family income. Under the demographic variables, duration of breastfeeding, age at marriage, sex of the previous child, parity and living status of the previous child, and use of contraceptive devices were considered. These variables were expected to cause the variation in the duration of PPA in view of the past findings. It was rather difficult to explore the effects of the explanatory variables in isolation owing to the fact that these were purely interdependent on one another. Here, the adopted tools had to quantify the possible effects of the variables after adjusting the effects of other variables under consideration.

### Methods of analysis

As the study was confined in the censored data, survival analysis techniques were used for statistical analysis. The Log-rank test was employed to compare the survival experience between different groups under study. The Cox's proportional hazard model was used for determining the effects of breastfeeding variables and other demographic and socioeconomic factors on the duration of PPA. The simple form of the Cox's proportional hazard model was given by λ (t;x)=λ0(t) exp (β/, x), where λ0(t) is the baseline hazard function and β is a vector of unknown regression coefficients to be estimated (15). The ratio—λ (t;x)/λ0(t)=exp (β/,x)—represents the ‘relative risk’ of failure or so termed as the risk of resumption of menstruation. Therefore, the hazard function enables one to estimate the relative risks of other groups in relation to the baseline group (reference group). When there is no covariate present in the model, exp (β/, x) is unity. The results are expressed in terms of β-coefficients, standard error (SE), p values, and relative risk (RR: eβ) with their 95% confidence interval (CI). The value of relative risk of a group of the study subjects greater than unity means that the risk of resumption of menstruation is higher for this group when compared with the reference group while values of less than one indicate that the risk is lower for the case group being analyzed when compared with the baseline group.

## RESULTS

### Univariate analysis

Table 1 shows the median durations of PPA according to the specific demographic and socioeconomic characteristics of the study women. The median duration of PPA in the study subjects was 5.7 months. The median duration of PPA increased with the increase in the duration of breastfeeding. By the Log-rank test, the association between the duration of breastfeeding and PPA was highly significant (χ^2^=41.439, p<0.01). A negative and strong association was found between the duration of PPA and the age at marriage of women (p<0.01). The median duration of PPA increased with the increase in parity, and this variation was highly significant (p<0.01) in the study subjects, irrespective of other covariates. The sex of the previous child did not influence PPA (p>0.05). The survival status of the previous child showed a strong effect on the duration of PPA (p<0.01). The longer duration of PPA was found with mothers who had an alive child (5.8 months) than who experienced a dead child (4.2 months). The duration of PPA for the couple who used contraceptive devices was significantly shorter than those who had never used any form of contraceptive devices (p<0.05). Non-users resumed menses six months after childbirth while users resumed menses after 5.3 months. The variation in the median duration of PPA according to educational level of couple was a downward linear trend, which was again highly significant (p<0.01).

### Multivariate analysis

To assess the partial effects of the explanatory variables on the duration of PPA while controlling for other covariates, multivariate proportional hazard model analysis was performed. The results are presented in Table 2. After the adjustment of other covariates under study, the duration of breastfeeding had a negative association with the risk of return to menses (β=-0.015, p<0.01). In fact, for every one-month increase in the duration of breastfeeding, the risk of resumption of menses decreased by 1.5% (RR=0.985). The survival status of the previous child during infancy had a significant effect (p<0.01) on the duration of PPA. The risk of resumption of menses of women with the survival of the previous child was 0.58 times lower than those who experienced the death of the previous child during infancy (RR=0.584). Among the socioeconomic variables, the place of residence had a significant effect (p<0.05) on the risk to return of menstruation after controlling for all other explanatory variables. The rural women were found to be subject to a hazard of menstruation 0.835 lower than that of urban women (RR=0.835).

## DISCUSSION

The findings showed that the median duration of PPA was 5.7 months. The findings also highlighted that the longer duration of breastfeeding was significantly associated with a lower risk of menstruation. This is due to the fact that prolonged lactation suppresses the production of certain types of hormones, thereby extending the postpartum anovulatory period. In fact, longer breastfeeding has suckling stimulation and proper prolactin secretion by the pituitary gland. Prolactin again suppresses gonadotropin (GnRH), which delays the secretion of follicle stimulation hormone (FSH) and Luteinizing hormone (LH), resulting in longer PPA. The results of this study are consistent with findings of studies in different countries (8-12, 16-18). The death of the index child during infancy was associated with the short duration of PPA, which is also consistent with findings of other studies (19,20). It may be possible that the death of a child during infancy cuts short the duration of nursing, which results in earlier resumption of menses and ovulation. The finding of the present study is also in the same direction.

The effects of age at marriage, parity, sex of the previous child, use of contraceptive devices, and parity were not at all significant. The place of residence had a significant relationship with the duration of PPA. The risk of resumption of menses for urban women was higher than rural women. The shorter duration of PPA among urban women was perhaps due to the fact that the educational level and employment status were higher among urban women than among rural women. In other words, the socioeconomic status of rural women was comparatively low due to their low level of education and employment. In contrast, most urban employed mothers did not get enough time to breastfeed their children as they work outside and, thus, tend to lactate for a shorter period and also probably provide food supplements to their children much earlier.

**Table 1.** Univariate analysis of duration of postpartum amenorrhoea

Variable	Median of PPA (months)	Log-rank test (χ^2^)
Demographic		
Duration (months) of breastfeeding		41.439, p<0.01
<5	4.8	
5-10	5.1	
10-15	5.8	
15-20	6.2	
20-25	6.8	
≥25	8.4	
Age (years) at marriage of wife		23.705, p<0.01
<15	6.4	
15-20	5.8	
20-25	5.1	
25-30	4.5	
≥30	4.3	
Parity		23.068, p<0.01
0	4.3	
1	5.1	
2	5.8	
3	6.4	
4+	6.9	
Sex of previous child		0.334, p>0.05
Female	5.1	
Male	6.2	
Survival status of previous child		8.340, p<0.01
Death	4.2	
Survival	5.8	
Use of contraceptives		5.453, p<0.05
No	6.0	
Yes	5.3	
Socioeconomic factors		
Place of residence		5.612, p<0.05
Urban	5.2	
Rural	6.0	
Educational level of husband		16.672, p<0.01
No schooling	6.9	
Primary school	6.0	
Seondary school	5.6	
Higher seondary school	5.2	
College and university	4.6	
Educational level of wife		15.403, p<0.01
No schooling	6.8	
Primary school	6.1	
Seondary school	5.7	
Higher seondary school	5.5	
College and university	4.9	
Family income (Rs)		7.306, p<0.05
<2,000	6.4	
2,000-<4,000	6.0	
4,000-<6,000	5.7	
6,000-<8,000	5.3	
8,000-<10,000	4.8	
≥10,000	4.3	
Overall	5.7	

PPA=Postpartum amenorrhoea

**Table 2.** Cox's multivariate regression analysis of duration of postpartum amenorrhoea

Variable	Coefficient (β)	Wald test (p value)	Relative risk (eβ)	95% CI for eβ
Demographic				
Duration (months) of breastfeeding	-0.015	11.703, p<0.01	0.985	0.976-0.994
Age (years) at marriage of wife	0.011	1.588, p>0.05	1.011	0.994-1.029
Parity	-0.027	2.393, p>0.05	0.973	0.940-1.007
Sex of previous child	-0.003	0.003, p>0.05	0.997	0.890-1.117
Survival status of previous child	-0.538	8.167, p<0.01	0.584	0.390-0.845
Use of contraceptives	-0.008	0.011, p>0.05	0.992	0.859-1.146
Socioeconomic				
Place of residence	-0.180	5.710, p<0.05	0.835	0.721-0.968
Educational level of husband	0.004	0.313, p>0.05	1.004	0.989-1.019
Educational level of wife	-0.005	0.465, p>0.05	0.995	0.980-1.010
Family income (Rs)	0.000	0.727, p>0.05	0.999	0.998-1.001

CI=Confidence interval

This finding is in agreement with findings of Srinivasan *et al*. (2), Aguirre *et al*. (6), and Aryal (7). Despite the importance of parity in the determination of the amenorrhoeic period, we found that this variable was not at all significant. It seems that its effect is absorbed totally by other related variables. This finding is contradictory with findings of other studies (3,7,10).

The findings of this interpretative study confirm that PPA is highly influenced by the duration of breastfeeding. Breastfeeding is again associated with other explanatory variables in many ways in the study subjects due to the diverse sociocultural and behavioural aspects. The policy-makers and planners, particularly maternal health planners are thus, suggested to promote breastfeeding practice and its associated factors to reduce fertility in the study subjects through prolonged birth intervals.

### Conclusions

The factors—breastfeeding, infant mortality, and residence―were found to have their high impacts on the duration of PPA. Prolonged breastfeeding can delay the resumption of menses following a livebirth, leading to lengthening of the subsequent birth intervals to reduce total fertility to achieve the national goal of replacement fertility of 2.1. Death of infants cuts down the nursing period, resulting in early resumption of menses and ovulation. Besides, evidence of high risk of short PPA among urban women might have been associated with higher education and employment, which are again linked to reduce the breastfeeding period. Hence, population scientists and health planners are needed to improve the factors associated with the prolonged duration of breastfeeding so that the eligible mothers could extend the duration of PPA.
